# ITS Polymorphism and Population Genomics Clarify the Relationship Between Group I and Group V in *Lentinula edodes*

**DOI:** 10.3390/jof12100735

**Published:** 2026-10-01

**Authors:** Ping Liu, Yuanhao Ma, Ling Hu, Xinru Zhao, Jingqiu Deng, Weimin Chen, Nan Tao, Hongmei Chai

**Affiliations:** Biotechnology and Germplasm Resources Research Institute, Yunnan Academy of Agricultural Sciences, Kunming 650205, China; 6771105002@lamduan.mfu.ac.th (P.L.); jianxyjian@126.com (Y.M.); 19887019544@163.com (L.H.); 18343190184@163.com (X.Z.); 13320580046@163.com (J.D.); chwmkm@aliyun.com (W.C.); tn1953@126.com (N.T.)

**Keywords:** Xianggu, haplotype, monokaryotization, single-spore, mating-type

## Abstract

Wild *Lentinula edodes*, also known as “Shiitake”, is distributed in many provinces of China, and southwestern China has the greatest genetic diversity of *L. edodes* germplasm resources. As determined by phylogenetic analyses of the internal transcribed spacer (ITS) of ribosomal RNA (rRNA), *L. edodes* was resolved into two non-sister lineages, known as Group I and Group V. In this study, the ITS fragments of 44 wild *L. edodes* strains, mainly from Yunnan Province of southwestern China, were cloned, sequenced, and analyzed. It was revealed that eight strains contained both Group I and Group V ITS. The protoplast monokaryotization strains (PMSs) and single-spore isolates (SSIs) were obtained from YAASM2342, whose mating types and ITS types were analyzed. The results confirmed that two heterokaryons of YAASM2342 contained Group I and Group V ITS, respectively. The strains of both Group I and Group V were present in the SSIs of each mating type at a ratio of ~1:1. Moreover, analyses of the population genomic dataset suggested that the recovered genetic groups could not correspond to Group I and Group V.

## 1. Introduction

*Lentinula edodes*, also known as “Shiitake” and “Xianggu”, is an edible and medicinal mushroom species. Wild populations of *L. edodes* are found in Asia, Australia, and the Americas [1], and have been recorded in 28 provinces and municipalities of China [2]. Moreover, regions of northwestern and southwestern China have the greatest genetic diversity of *L. edodes* germplasm resources [3,4]. The artificial cultivation of *L. edodes* dates back more than 800 years, and the cultivated strains have been domesticated so that they have superior quality for human consumption. Since 2018, in Yunnan Province, located in southwestern China, some wild strains of *L. edodes* were directly used for commercial cultivation, and the harvested fruiting bodies were called “small Xianggu”, which is distinguished by a thin-fleshed, smooth pileus and slender stipe, and mild thiol-type odor, and represent a group with conspicuous morphological characteristics. The scale of cultivation of “small Xianggu” is expanding.

Based on the phylogenetic analysis of ITS, *L. edodes* was divided into two non-sister lineages, which were called Group I and Group V [5]. Group I was subdivided into Groups Ia and Ib [4,5]. Strains of Group Ia were mainly found in northeastern Asia, and strains of Group Ib were distributed in Pan-mid-southern Asia. Group V is a typical highland-type group whose strains occur in western China, India, and Nepal. Moreover, the strains of Group Ia, Group Ib, and Group V overlap in southwestern China, represented by Yunnan, Guizhou, and Sichuan provinces [4,6].

However, both separate analyses of translation elongation factor (*tef1*-α) and concatenated analyses of ITS + LSU + *tef1-α* resolved only one lineage for Group I and Group V [7]. Population genomic analyses performed by Sierra-Patev et al. [8] revealed that *L. edodes* sensu lato (s. lat.) contains three lineages that may be identified as species, and Group I and Group V, recovered by ITS, have been retained as polymorphisms within *L. edodes* s. lat. The clades based on genome-wide SNPs did not correspond to Group I and Group V, and this discordance may occur due to the polymorphisms in rRNA genes [7].

Intra-strains of *L. edodes* exhibit high-level polymorphisms in ITS regions [9,10], and direct PCR and sequencing of most strains often yield chromatographs with many heterozygous sites, which introduce inaccuracies into classification. Internal transcribed spacer (ITS) polymorphisms of 88 *L. edodes* strains were investigated by Song et al. [10]. They divided the ITS1 sequences into a and b types, which contained 26 haplotypes. The ITS2 sequences, including 24 haplotypes, were subdivided into a1, a2, b1, and b2 types.

The primary goal with this study is to investigate the causes of ITS polymorphism within strains and clarify the relationship between Group I and Group V in *L. edodes*. The ITS fragments of 44 wild strains of *L. edodes*, which are mainly from southwestern China and include 10 strains of “small Xianggu”, were cloned, sequenced, and analyzed. The inheritance patterns of divergent ITS sequences were further characterized by examining their distribution among single-spore isolates from YAASM2342, a heterozygous strain. Moreover, population genome analyses were performed to assess the status of Group I and Group V of *L. edodes*.

## 2. Materials and Methods

### 2.1. Strains and Cultivation

A total of 44 wild strains of *L. edodes* from China were used in this study (Table S1). Six strains, denoted as ZAASLY, were provided by the Horticulture Research Institute, Zhejiang Academy of Agricultural Sciences, and strain LE0048 was provided by the Soil and Fertilizer Research Institute, Guizhou Academy of Agricultural Sciences. Moreover, 10 strains, noted as “small Xianggu”, were isolated from different cultivation sites in Yunnan Province. The other strains were isolated from wild specimens collected from remote mountain areas. Strain isolation was made immediately on the day of collection, and the isolates were transported to the laboratory for incubation. All strains were stored in the National Collection of Edible Fungi (Yunnan).

An agar disk (5 mm in diameter) containing mycelia was transferred to a Petri dish containing yeast extract peptone dextrose (YPD) medium, and the plate was maintained for 14 days in the dark at 23 °C. Then, mycelia were scraped, frozen in liquid nitrogen, and used for DNA extraction.

The YAASM2342 strain was cultivated by Yunnan Junsi Biotechnology Co., Ltd (Jinnin county, Yunnan Province, China) The cultivation substrates contained 2000 g of medium, comprising 80% sawdust, 18% bran, and 2% gypsum. From the harvested offspring, a mature fruiting body was randomly collected to obtain a spore print.

### 2.2. DNA Extraction, Amplification, Cloning, and Sequencing of ITS Regions

Initially, total DNA was isolated from mycelia using the Fungal gDNA Isolation Kit (BW-GD2416, Beiwo Biotech Co., Ltd., Hangzhou, China). Next, the fungal universal primer pairs ITS1 and ITS4 were used for amplifying the ITS [11]. The PCR reaction mixture (50 µL) contained 25 µL of 2 × PCR Mix (Vazyme Biotech Co., Ltd., Nanjing, China), 1 µL of each 10 mM primer, and 1 µL of 10 ng/µL genomic DNA. The cycling conditions were as follows: denaturation at 95 °C for 3 min, followed by 30 cycles at 95 °C for 30 s, 57 °C for 30 s, and 72 °C for 60 s. The final extension was performed at 72 °C for 10 min [12].

After the PCR products were purified using a *Trelief* DNA Gel Extraction Kit (Dalin Bio-tek. Inc., Beijing, China), they were cloned in *Escherichia coli* DH5 using the pClone007-T versatile Simple Vector Kit (Tsingke Biotech Co., Ltd., Beijing, China). Ten to eleven monoclonal colonies for each strain were sent to Beijing Tsingke Biotechnology for Sanger sequencing by ABI-3730xl using T-vector universal primer pairs M13F/R for bidirectional sequencing, while fewer than ten positive clones were obtained for strains YAASM397, YAASM3538, and YAASM7662. For strains YAASM126, YAASM418, YAASM1133, YAASM1518, and YAASM 2342, some ambiguous sequences were detected after analysis, and re-amplification and cloning experiments were repeated, resulting in a total of 22 to 30 clone sequences subjected to subsequent analysis (Table S1). Altogether, 518 cloned ITS sequences were obtained from the 44 strains.

### 2.3. Alignment and Phylogenetic Analyses of ITS Sequences

After manually removing vector sequences at both ends, the ITS sequences were assembled and trimmed using SeqMan Pro in DNASTAR 7.1.0. By aligning with the ITS sequences of *L. edodes* [10] using ClustalX 1.83, the ITS1 and ITS2 sequences were delimited, and the haplotype of each sequence was determined. In total, 32 ITS sequences of *Lentinula* were downloaded from the GenBank nucleotide database (Table S2). Based on the Akaike Information Criterion (AIC) [13], the optimal nucleotide substitution model HKY + I was selected using jModelTest 2.1.10 [14,15]. Next, Bayesian Inference (BI) phylogenetic trees were constructed using MrBayes 3.2.7 [16], with *L.* aff. *lateritia* designated as the outgroup [7]. Four independent runs were conducted, each containing four chains over 20,000,000 generations, with posterior sampling every 1000 generations. The initial 25% of sampled trees were removed as burn-in; Bayesian posterior probabilities of clades were estimated from the remaining trees. Convergence was reached if the average standard deviation of split frequencies was less than 0.01. The phylogenetic trees were edited using FigTree 1.4.4 and Adobe Illustrator 25.3.1.

### 2.4. Isolation of Strains of Protoplast Monokaryotization and Single Spores

The protoplast preparation method was referred to Tao et al. [17], with certain modifications. Fully grown mycelia from the YAASM2342 strain were inoculated in 25 mL of YPD liquid medium and incubated at 23 °C. After seven days, the mycelia were collected by filtration through 200-mesh gauze and rinsed three times with 0.6 M mannitol. Then, they were suspended in 0.6 M mannitol containing lytic enzymes (2% lywallzyme (Institute of Microbiology, Guangdong Academy of Sciences, Guangzhou, China), 2% cellulase, and 2% snailase (Solarbio Biotech Co., Ltd., Beijing, China) and incubated at 30 °C for 2 h. Protoplasts were separated from hyphal debris by filtration through 500-mesh gauze, washed twice with 0.6 M mannitol, and collected by centrifugation at 3000 rpm for 15 min at 4 °C. For regeneration, serially diluted protoplasts were plated on YPD medium containing 0.6 M sucrose and incubated at 23 °C until the formation of new colonies. Viable colonies were transferred to new PDA plates, and protoplast monokaryotization strains (PMSs) without clamp connection were verified under a microscope (DM6 B, Leica Microsystems IR GmbH, Wetzlar, Germany).

To isolate single-spore strains, a spore suspension was prepared by serial dilution, and a 100 µL suspension was spread on PDA plates. Then, the plates were incubated at 23 °C. Growing colonies were observed under a microscope, and single-spore isolates (SSIs) without clamp cells were placed onto new PDA slants.

### 2.5. Determination of the Mating Type of Monokaryotic Strains

The primer pairs AF/AR for the *A* mating-type loci and BF/BR for the *B* mating-type loci were designed based on the primers reported by Zhang et al. [18] (Table S3). Standard PCR analysis was conducted to examine the monokaryotic populations from the YAASM2342 strain, after which the PCR products were sequenced.

Multiple-sequence alignment analyses of the *A* and *B* mating-type loci were performed, respectively, using the software ClustaIX 1.83, and the differences between the mating-type alleles were checked. In this study, one allelic sequence of the *A* loci was designated as *A1*, and another allele was designated as *A2*; accordingly, *B1* and *B2* of the *B* loci were confirmed. Next, the allele of each monokaryotic strain was counted, and consequently, the mating type was determined.

Confrontational tests between the monokaryotic strains were conducted to assess the accuracy of the mating types. Next, 2–3 strains were randomly selected from each mating-type group to test mycelial interactions with members within groups and between groups, and mycelia from each side of the pairs were examined for clamp connections. Additionally, phenotypic features of the antagonistic reaction were observed and recorded.

### 2.6. Genome Re-Sequencing Analysis

Qualified DNA samples were sent to Shanghai Personal Biotechnology Co., Ltd. (Shanghai, China), and the library was prepared following the standard process for building libraries using the TruSeq DNA PCR Free Prep Kit reagent (Illumina, San Diego, CA, USA). The samples were sequenced on the Illumina NovaSeq platform with 150 bp paired-end and a 400 bp insert size, and the raw data were filtered using fastp (v0.20.0) by scanning reads with a sliding window.

All clean reads were aligned to the reference genome (haploid strain L808 of *L. edodes* (NCBI accession no. GCA_015476405.1)) using BWA 0.7.17 [19] with the default settings. Single nucleotide polymorphisms (SNPs) were identified by GATK [20] and only bi-allelic SNPs with <20% missing data and a minor allele frequency (MAF) > 0.05 were retained for further analysis.

Principal component analysis (PCA) was conducted using PLINK (v1.9) [21]. An approximately maximum-likelihood phylogenetic tree was inferred from the SNP alignment using FastTree v2.1.11 [22] under the GTR nucleotide substitution model. Tree optimization used the default CAT approximation with 20 rate categories, followed by branch-length rescaling and likelihood calculation under the Gamma20 model (-nt -gtr -gamma). Branch support was evaluated using SH-like local support values based on 1000 resamplings of site likelihoods. To investigate the population structure, the number of genetic clusters present in the genomes of the population was determined using K from 2 to 10 with ADMIXTURE 1.3.0 [23]. Then, a cross-validation (CV) procedure in ADMIXTURE was used to estimate the number of ancestral populations (*K*).

## 3. Results

### 3.1. ITS Polymorphism of L. edodes

In total, 518 cloned ITS sequences (Table S1) were obtained from 44 wild *L. edodes* strains. The haplotypes of ITS1 and ITS2 were identified, respectively (Table S1) by aligning them with the sequences submitted by Song et al. [10]. Some new haplotypes of ITS1 (Hap27 and Hap28) and ITS2 (Hap25 to Hap32) were identified (Figure S1, Table S4).

Haplotypes of ITS1 and ITS2 were arranged in tandem for each ITS sequence, and 32 haplotype combinations were found in the 44 strains. Based on ITS1 (a/b) and ITS2 (a1/a2/b1/b2) [10], these 32 haplotype combinations could be included in seven ITS types as follows: aa1, aa2, bb1, bb2, ab1, ab2, and ba2; nevertheless, follow-up experiments confirmed that ab1, ab2, and ba2 types represented chimeric artifacts instead of the genuine endogenous sequences of the tested strains. A phylogenetic tree was constructed using ITS sequences extracted from the 32 haplotype combinations (Figure 1). Four subclades were found in the Group I lineage. The sequences of the ITS-aa1 type nested in Group Ia included isolates from Japan, Russia, and isolates from northeastern, northwestern, and southwestern provinces of China. The ITS-aa2 sequences were clustered with isolates from the eastern and central provinces of China and formed Group Ib. The ITS-ba2 and ITS-ab sequences were clustered, respectively, and identified as two subclades of Group I. The ITS-bb sequences were placed in the Group V lineage, which included isolates from Nepal, Vietnam, and China.

### 3.2. ITS Heterogeneity Within L. edodes Individuals

There were 34 homogeneous ITS individuals among the 44 strains (Table 1), and the other 10 strains were in a heterozygous state; six strains among them had two types of ITS sequences each. The strains YAASM114 and YAASM734 contained ITS-bb1 and ITS-bb2, and could be classified into Group V. The strains YAASM1515, YAASM7604, and YAASM7607 each had ITS-aa and ITS-bb, i.e., each strain had both Group I and Group V ITS. Similar strains were also reported by Song et al. [10]. Among the *L. edodes* strains in their study, nine strains were of the ITS-aa and ITS-bb type, while one strain was of the ITS-aa2 and ITS-ba2 type.

Five strains in this study were found with four ITS types each. The strains YAASM126, YAASM1133, and YAASM1518 each contained aa2, bb1, ab1, and ba2 sequences. The strains YAASM418 and YAASM2342 both possessed aa2, bb2, ab2, and ba2 sequences. *Lentinula edodes* is heterokaryotic, and the two nuclei with different ITS sequences may be the main reason that two types of ITS are present in a strain. However, why a strain has four different types of ITS is not clear.

### 3.3. ITS Polymorphic Distribution Within Different Mating-Type Monokaryotic Isolates from Strain YAASM2342

The strain YAASM2342 with four ITS types (aa2, bb2, ab2, and ba2) was used to investigate the composition and inheritance patterns of the divergent ITS sequence, and some PMSs and SSIs were obtained. Among 110 regenerated isolates from protoplasts, only 10 isolates were checked for no clamp connection and were therefore monokaryotic isolates. Additionally, 77 SSIs were obtained via gradient dilution. The mating type of these monokaryotic isolates was determined based on SNP genotyping obtained by using two primer pairs, AF/AR and BF/BR. In the obtained *A* mating-type fragment (about 500 bp), a total of seven allelic SNP variations were observed (Figure S2). For the *B* mating-type sequence (about 1100 bp), 12 SNPs and three indels (3 bp,1 bp, and 1 bp, respectively) were detected (Figure S3). The allelic genotypes of the *A* and *B* mating factors from PMS-45 were designated as *A1* and *B1*, respectively, and the alternative allelic genotypes were correspondingly *A2* and *B2*. Finally, seven PMSs, including PMS-45, were determined as mating-type *A1B1*, and the other three PMSs, including PMS-67, were mating-type *A2B2*. For the 77 SSIs, the numbers for mating types *A1B1*, *A2B2*, *A1B2*, and *A2B1* were 16, 24, 13, and 24, respectively (Table S5).

To assess the accuracy of the mating types based on SNP genotyping, 2–3 monokaryotic isolates were randomly selected from each mating-type group to test their confrontational interactions with members within a group and between groups. Clamp connection was observed between isolates with different *A* and *B* factors. When the paired strains had the same *A* or *B* factor, typical or atypical barrages formed along the contact zone, and no clamp connection was found (Figure S4, Table S6). The results of the confrontation tests confirmed the correctness of the mating type of the monokaryotic isolates.

The ITS type analyses were conducted among 58 monokaryotic isolates, including 10 PMSs and 12 SSIs for each mating type. All PCR products were sequenced directly, and the chromatograms of the ITS regions did not show double peaks. The sequence alignment analyses revealed that the seven PMSs of mating-type *A1B1* possessed ITS-bb2, whereas the other three PMSs of mating-type *A2B2* possessed ITS-aa2. Among the 12 SSIs of each mating type, there were both isolates of ITS-bb2 and ITS-aa2, and the ratio was approximately 1:1 (Figure 2).

### 3.4. Ambiguous ITS Sequences Arising from PCR Chimeras

Surprisingly, no ITS-ba2 or ITS-ab2 was detected in the monokaryotic isolates of YAASM2342; therefore, the ITS regions of the 10 PMSs were cloned and analyzed again, and 10 positive colonies for each isolate were sequenced. According to the results, no ITS-ba2 or ITS-ab2 were found in these 100 cloned ITS sequences, and the 10 cloned sequences of each PMS were almost identical.

Two templates of heterologous genomic DNA were simulated by mixing equal amounts of DNA from PMS-63 (ITS-bb2) with PMS-67 (ITS-aa2), and from PMS-95 (ITS-bb2) with PMS-97 (ITS-aa2). Among the 20 cloned sequences obtained from each template, one ITS-ab2 sequence and one ITS-ba2 sequence were identified (Figure S5). These findings confirmed that ITS-ab2 and ITS-ba2 from the strain YAASM2342 are chimeric sequences generated during PCR amplification using heterologous DNA templates.

Among the 44 strains in this study, none had only the ITS-ab or ITS-ba sequence. Additionally, the ITS-ab and ITS-ba sequences were present at low abundance among the cloned sequences of each strain (Table S1). Accordingly, ITS-ab2 and ITS-ba2 from the other strains were considered to be chimeric amplicons.

### 3.5. Genome Re-Sequencing and Diversity

Genome re-sequencing was performed, including 44 wild *L. edodes* strains and six monokaryotic isolates from YAASM2342 as follows: PMS-45 (ITS-bb2/*A1B1*), PMS-67 (ITS-aa2/*A2B2*), SSI-60 (ITS-aa2/*A1B1*), SSI-144 (ITS-aa2/*A1B2*), SSI-55 (ITS-bb2/*A2B1*), and SSI-90 (ITS-bb2/*A2B2*).

At least 2 Gb of clean DNA reads with Q30 > 94% were obtained for each strain and mapped to the reference genome L808. The mean nominal sequencing depth, calculated as the sequencing yield divided by the reference genome size, was 48.7×, and an average of 89.5% of the reference genome was covered by at least one read. The reads had an average mapping rate of 80.5% (Table S7). Among the SNPs dispersed throughout the genome, 1,436,459 SNPs with a MAF > 0.05 and <20% missing data were selected for analyzing population genomics. A total of 2,441,942 transition mutations (Ts) and 881,715 transversion mutations (Tv) were identified, with a Ts/Tv ratio of 2.90. The Ts frequency was 73.47%, whereas the Tv frequency was 26.53% (Figure S6).

### 3.6. Population Structure of L. edodes

An admixture analysis was conducted for the 50 strains of *L. edodes,* and four major populations were revealed when the lowest CV error was observed for K = 4 (Figure S7). Population 1 (Pop1) contained seven strains, including two from North China (Jilin Province), two from East China (Zhejiang and Fujian Provinces), and three from Southwest China (Yunnan and Guizhou Provinces). Seven “small Xianggu” strains constituted population 2 (Pop2), while YAASM2342 and its six monokaryotic isolates formed population 3 (Pop3). The remaining 29 strains formed population 4 (Pop4), which were from Yunnan Province and contained three “small Xianggu” strains (Figure S8). The PCA results were congruent with the results of the admixture analyses (Figure 3, Table S8). Pop1 and Pop2 included only strains of ITS-aa type, except for YAASM7604 (ITS-aa + ITS-bb), while Pop3 and Pop4 concentrated on the strains of three ITS types (ITS-aa, ITS-bb, and ITS-aa + ITS-bb).

In the ML tree constructed using SNPs, all wild strains of ITS-bb were clustered together. Four strains of the ITS-aa + ITS-bb type and one strain of the ITS-aa strain were distributed in this group. Except for three monokaryotic isolates of the ITS-bb type included in the branch of the parent YAASM2342 and its progeny strains, the other clades were strains of the ITS-aa and ITS-aa + ITS-bb types.

## 4. Discussion

### 4.1. ITS Barcode Is Problematic for the Phylogenetic Analysis of L. edodes

As ITS serves as a barcode for filamentous fungi, the predicted thresholds were generally 97% to 99% for species and 94% for genus [24]; nevertheless, ITS evolves at highly variable rates in different clades, which leads to clade-specific taxonomic thresholds [25]. The sequence similarities between different ITS types of *L. edodes* were 93.6–99%. In the phylogenetic analysis of the ITS data alone, *L. edodes* strains were resolved into two non-sister lineages, Group I and Group V [5], which were regarded as two phylogenetic species and were denoted as *L. edodes* and *L.* aff. *edodes* [7]. However, some wild strains that contained one Group I nucleus and one Group V nucleus were identified in this study, and they could produce offspring that could be classified as Group I, Group V, and heterozygous Group I + Group V. Therefore, intrastrain variations in the ITS region may obscure phylogenetic relationships.

Double or multiple peaks in chromatograms were frequently detected when ITS products of strains of *L. edodes* were directly sequenced, and this phenomenon was often ignored. However, clone sequencing in this study revealed that some chimeric sequences may be overrepresented when heterologous genomic DNA serves as the PCR template, which results in an overestimation of diversity. Chimera formation occurs more often in genetically diverse and species-rich samples [26]. By comparing the ITS cloned sequences generated from heterokaryons and homokaryons of *Agrocybe aegerita*, Qian et al. [27] found that chimeric sequences amplified from heterokaryons accounted for 30–41.67% of all clones, and the SNP and indel variations in these sequences were derived from template switching at specific loci. Therefore, when performing phylogenetic analyses using multi-copy regions, such as ITS, chimeric sequences generated during amplification need to be identified.

### 4.2. Two Nuclei with Divergent ITS Variations Are the Main Reason for ITS Polymorphisms Within Dikaryotic Strains of L. edodes

Variations in ITS within strains occur in some fungi, such as *Ophiocordyceps sinensis* [28], *Aspergillus nidulans* [29], and *Laetiporus* spp. [30], probably because of intragenomic variations (a variation that exists within the haploid genome between multiple copies of the same gene or region) [31]; however, for dikaryotic basidiomycetes, such variations probably occur due to the divergence between two haploid nuclei [32].

In total, 10 PMSs and 48 SSIs from YAASM2342 were detected; 7 PMSs of *A1B1* mating type were identified as Group V ITS, and the other 3 PMSs of mating-type *A2B2* were Group I ITS. Among the 48 SSIs, half exhibited Group V ITS and the other half displayed Group I ITS. These results suggest that the two nuclei of YAASM2342 harbored Group I and Group V ITS types, respectively. In the present study, eight strains possessed both Group I and Group V ITS types, and we infer that, analogous to strain YAASM2342, each of these strains contains two nuclei with Group I and Group V ITS types, respectively.

By analyzing 12 SSIs of each mating type (*A1B1*, *A2B2*, *A1B2*, and *A2B1*), we found that both Group I and Group V ITS were present in SSIs of each mating type, with an approximate ratio of 1:1. This suggested that the *A* and *B* mating factors and multiple copies of rDNA represent non-linkage inheritance and undergo independent assortment into daughter spores during meiosis. For the progeny of YAASM2342, the probability of obtaining dikaryotic strains of Group I, Group V, and heterozygote (Group I + Group V) via crossing was equal. In southwestern China, strains of Group I and Group V broadly overlap [4]; this also indicates that a certain number of heterogenetic strains, including Group I and V ITS, must be present in this area.

### 4.3. Population Genomics Revealed That Strains of Group I and Group V of L. edodes Could Not Be Resolved as Two Distinct Populations

Genome-wide population analyses on cultivated and wild *L. edodes* resolved three principal clades, wherein two clades mainly consisted of wild strains, while the other comprised admixed wild and cultivated strains [8,33]. However, Group I and Group V failed to correspond to the three clades identified via population genomic analyses, with their strains scattered across all clades [8].

In this study, population structure analyses showed that all Group V strains, a subset of Group I strains, and Group I + Group V strains co-clustered into one cohesive genetic population. Moreover, the three monokaryotic isolates of Group V were nested in Pop3 with their parent YAASM2342 and another monokaryotic isolate of Group I. No Group V-exclusive discrete population was detected at the whole-genome scale. In addition, when these 50 strains were classified into three groups (Group I, Group V, and Group I + Group V) based on their ITS types, all pairwise Fst values (0–0.05) (Table S9) revealed low genetic divergence across groups. These results indicated that strains of Group I and Group V could not be resolved as two distinct genetic populations.

However, the number of samples analyzed in this study was relatively small, and most strains were collected from Yunnan Province of China. Geographic representation and potential sampling bias imposed certain limitations on the present study. It is currently believed that strains distributed in Nepal and India predominantly belong to the Group V ITS type, whereas those from South Korea, Japan, and Northeast China are mainly the Group I ITS type [4,5]. Further investigation of more strains to determine whether or not the heterozygous strains harboring both Group I and Group V ITS types exist in other geographical regions will facilitate a better understanding of the genetic relationships between Group I and Group V strains.

## 5. Conclusions

Several strains of *L. edodes* containing both Group I and Group V ITS types were identified in this study. The study also found that ITS variations within these strains were due to the divergence between two haploid nuclei, and their progeny monosporous strains were homokaryons of either Group V or Group I. Accordingly, it is reasonable to infer that a certain number of Group I + Group V strains occur in nature. When heterologous genomic DNA serves as a PCR template, some chimeric ITS sequences may be formed, resulting in an overestimation of diversity. Moreover, population genomic data analysis confirmed that strains of Group I and Group V did not resolve into two independent groups.

## Figures and Tables

**Figure 1:**
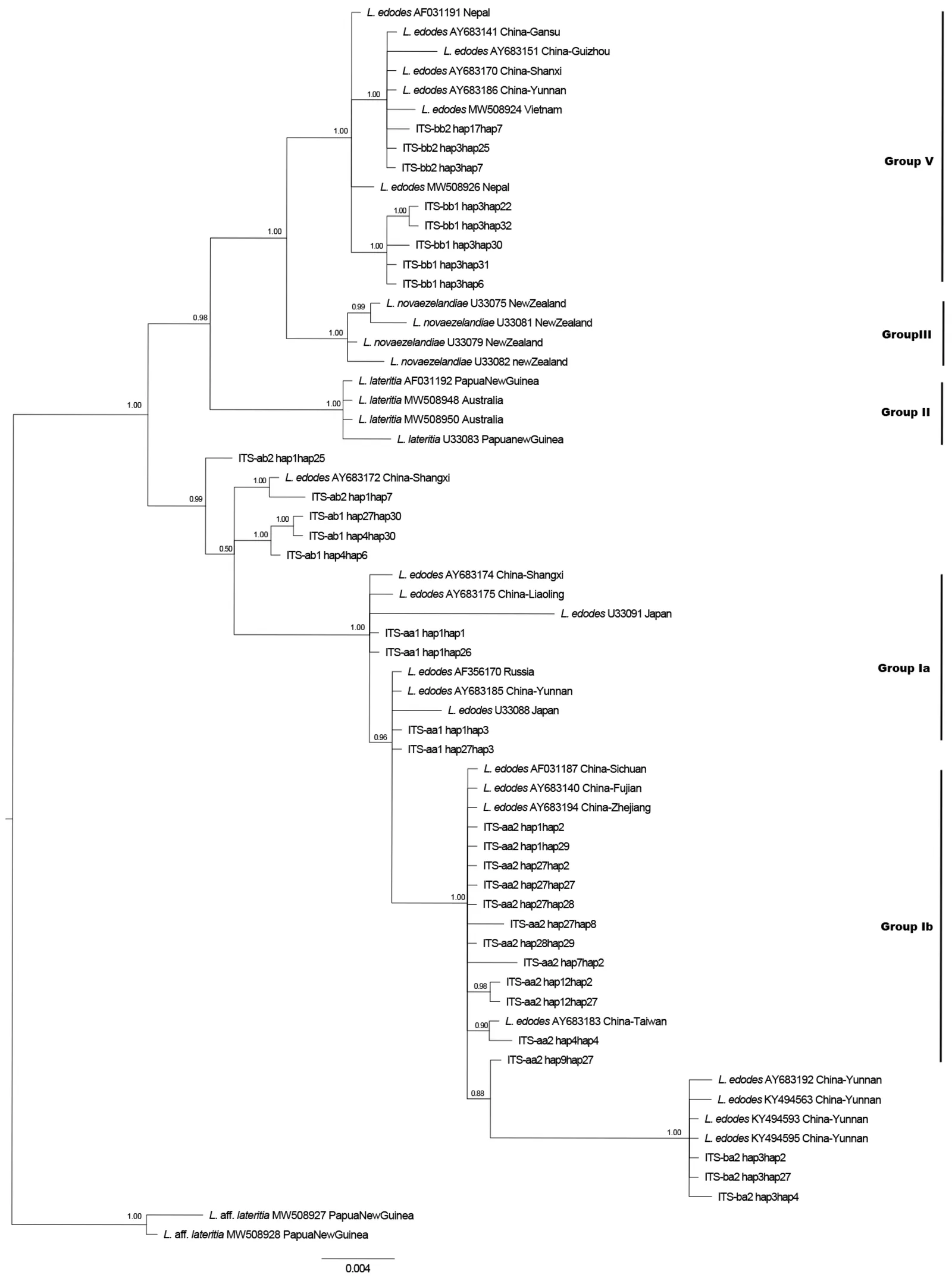
The BI tree was constructed based on the complete ITS sequences of 32 haplotype combinations from 44 *L. edodes* strains. Support values (BS > 50%) are shown at the nodes. Black vertical lines on the right indicate group boundaries.

**Figure 2:**
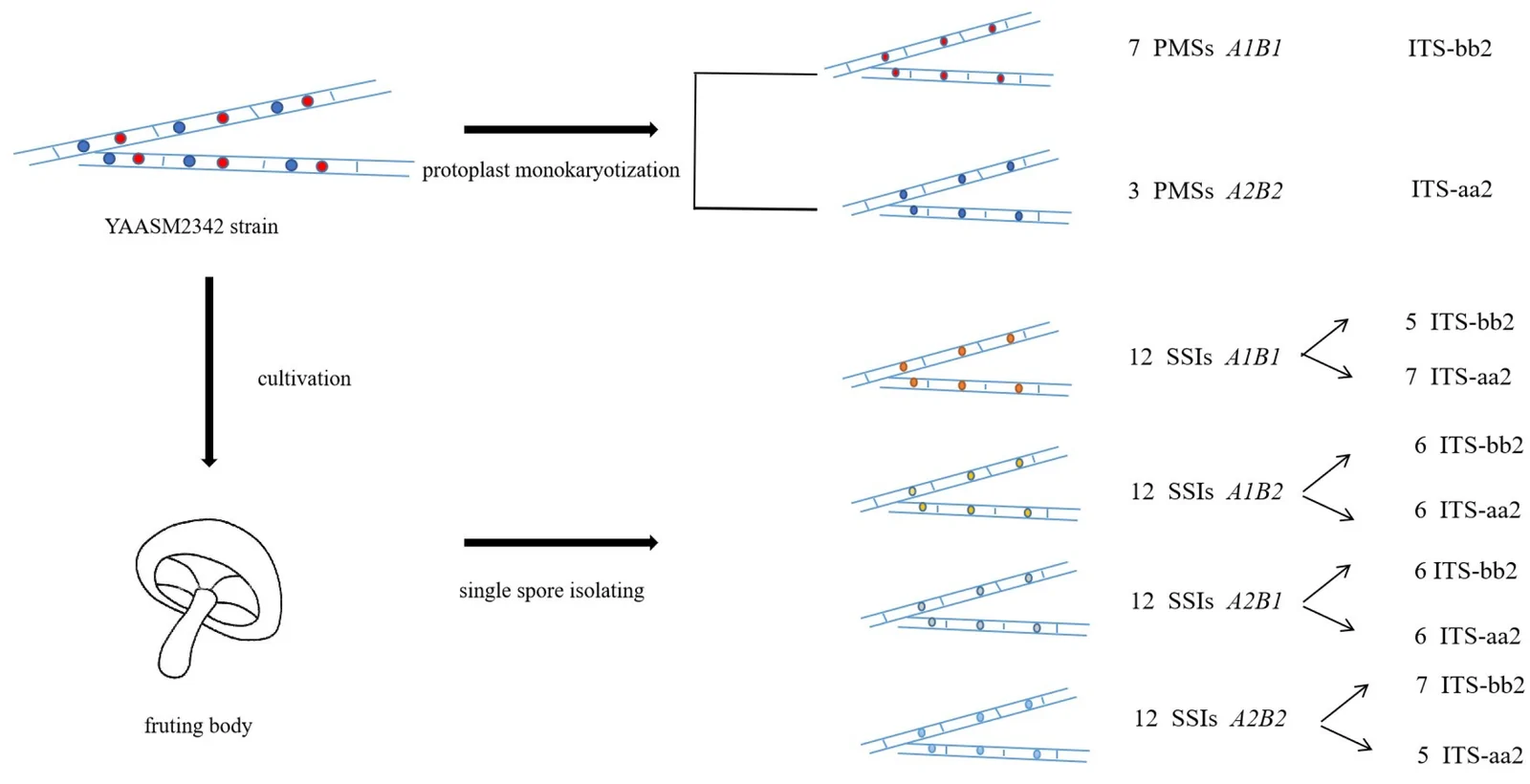
Schematic illustration of the ITS types and mating type of the monokaryotic population from YAASM2342.

**Figure 3:**
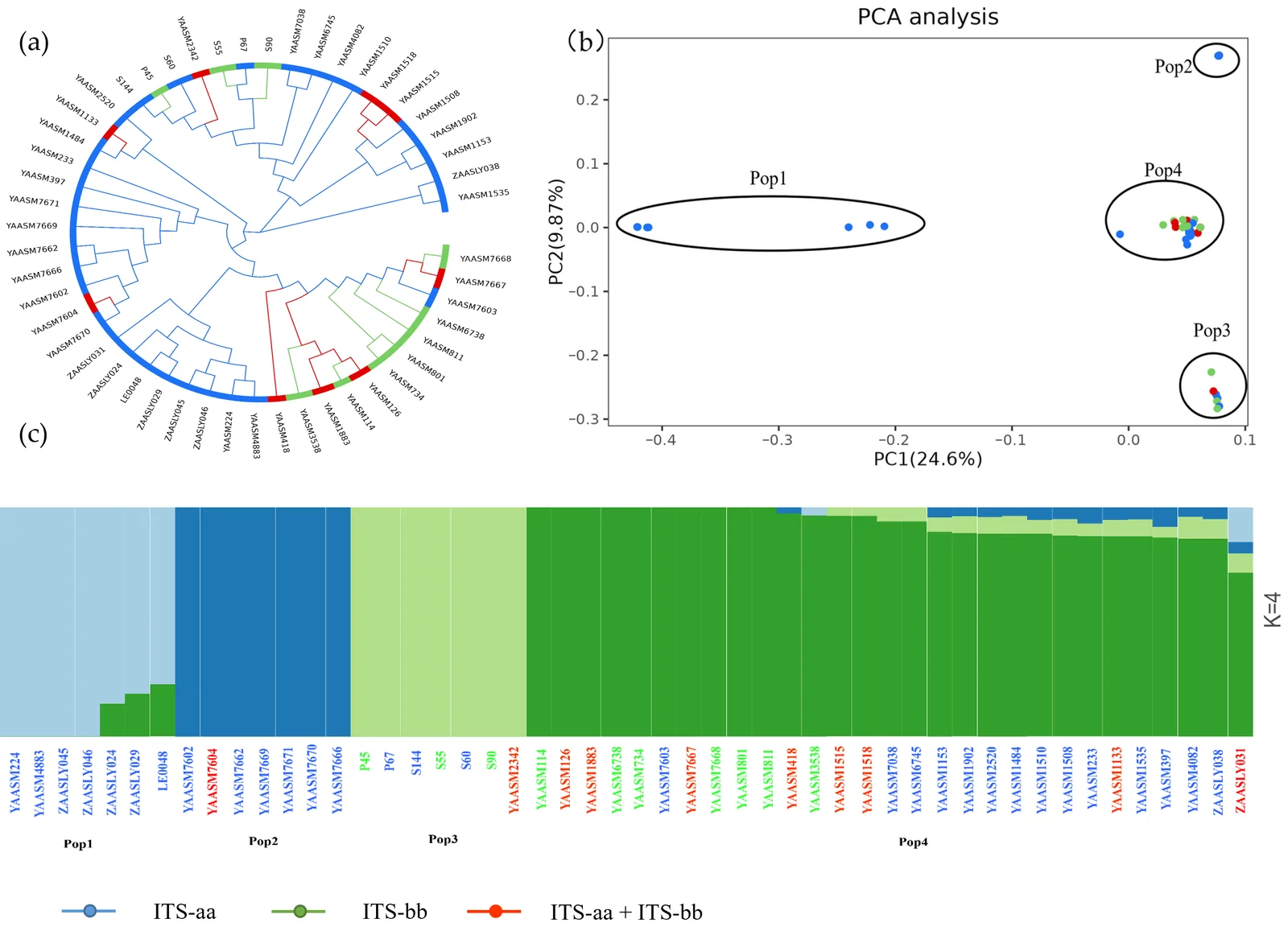
Population structure of 50 *L. edodes* strains based on 1,436,459 SNPs. (**a**) ML phylogenetic tree; (**b**) PCA; (**c**) admixture result at K = 4.

**Table 1:** List of the strains with different types of ITS.

Number	ITS Types Within Individual	Strain
1	aa1	YAASM224 YAASM4883 YAASM7645 YAASM7646
2	aa2	YAASM233 YAASM397 YAASM1153 YAASM1484 YAASM1508 YAASM1510 YAASM1535 YAASM1902 YAASM2520 YAASM4082 YAASM6745 YAASM7038 YAASM7602 YAASM7603 YAASM7640 YAASM7641 YAASM7642 YAASM7644 YAASM7649 YAASM7662 YAASM7666 YAASM7669 YAASM7670 YAASM7671
3	bb1	YAASM801 YAASM811 YAASM1883 YAASM3538
4	bb2	YAASM6738 YAASM7668
5	bb1 + bb2	YAASM114 YAASM734
6	aa2 + bb1	YAASM1515
7	aa2 + bb2	YAASM7604 YAASM7667
8	aa2 + bb1 + ab1 + ba2	YAASM126 YAASM1133 YAASM1518
9	aa2 + bb2 + ab2 + ba2	YAASM418 YAASM2342

## Data Availability

The original contribution presented in this study are included in the article/Supplementary Material. Further inquiries can be directed to the corresponding authors.

## Supplementary Materials

The following supporting information can be downloaded at: https://www.mdpi.com/article/10.3390/jof12100735/s1, Figure S1: ITS2 sequence alignments of the new haplotypes and the core haplotypes; Figure S2: Sequence alignments of the mating *A* loci from 10 PMSs of YAASM2342; Figure S3: Sequence alignments of the mating *B* loci from 10 PMSs of YAASM2342; Figure S4: Antagonism test of the monokaryotic strains of different mating types; Figure S5: Sequence alignments of the chimeric amplicons from mix template; Figure S6: Analyses of SNP mutation spectrum; Figure S7: Distribution map of CV error values to different K values; Figure S8: Distribution map of 50 *L. edodes* from four populations; Table S1: Summary of strains and the ITS types; Table S2: ITS sequences of the genus *Lentinula* were downloaded from the GenBank nucleotide database; Table S3: Primer sequences amplified the mating-type locus; Table S4: The new haplotypes in this study; Table S5: The mating types and the ITS types of the monokaryotic strains from YAASM2342; Table S6: Confrontation test of the monokaryotic strains from different mating-type groups; Table S7: Statistics of reads for each strain; Table S8: PCA data; Table S9: Fst results statistics among strains with different ITS types.
