# Supplementary material for: ITS Polymorphism and Population Genomics Clarify the Relationship Between Group I and Group V in Lentinula edodes
Source: J Fungi (Basel). 2026 Oct 1;12(10):735. doi: 10.3390/jof12100735 (PMC13705675; doi:10.3390/jof12100735)
Supplement: Supplementary file 1 [file jof-12-00735-s001.zip › Supplementary Figure.pdf]

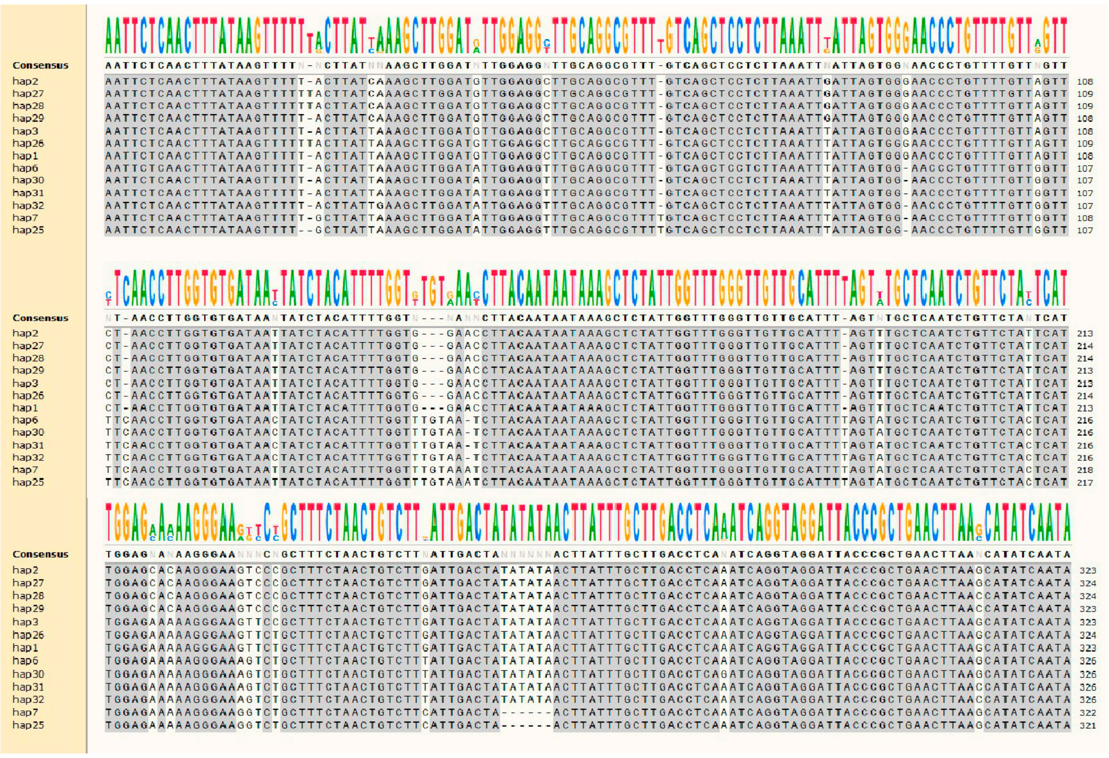


**Figure S1**. ITS2 sequence alignments of the new haplotypes and the core haplotypes. Hap25 to hap32 were new haplotypes identified in this study, and the other haplotypes were retrieved from Song *et al*. Hap1 and hap3 are the core haplotypes of ITS2-a1; hap2 belongs to ITS2-a2, hap6 to ITS2-b1, and hap7 to ITS2-b2.

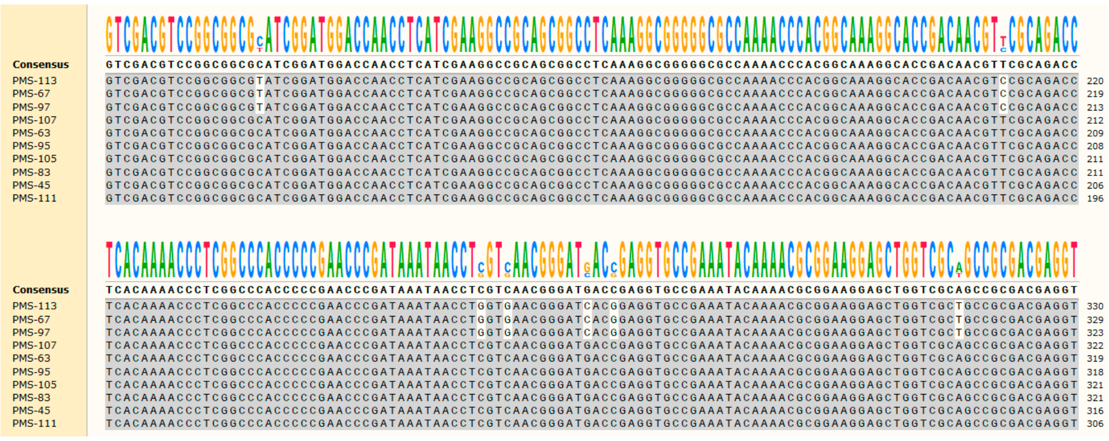


**Figure S2.** Sequence alignments of the mating *A* loci from 10 PMSs of YAASM2342. Seven monokaryotic strains including PMS-45 belong to the *A1* mating-type, and the other three monokaryotic strains including PMS-67 are the *A2* mating-type.

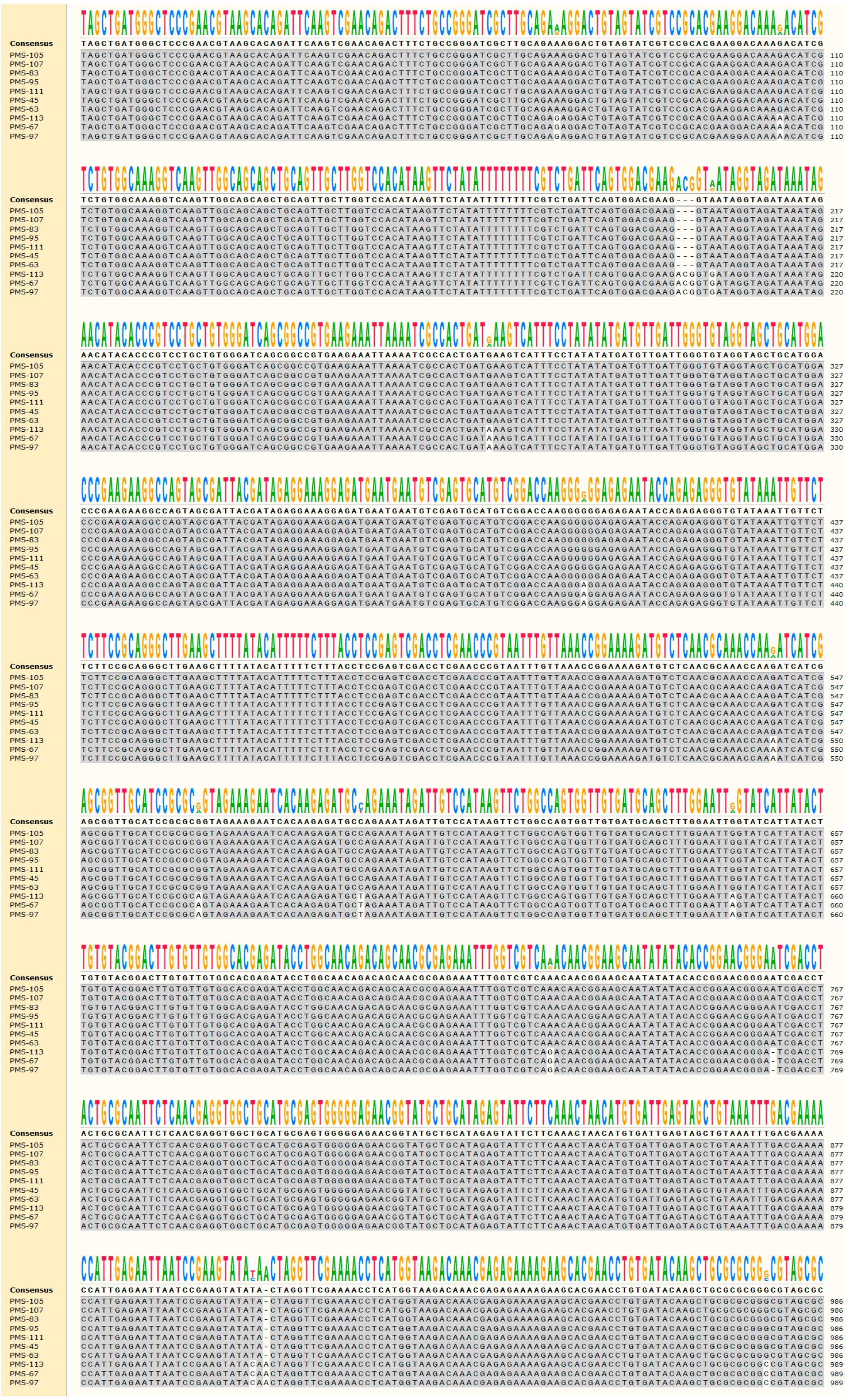


**Figure S3.** Sequence alignments of the mating *B* loci from 10 PMSs of YAASM2342. Seven monokaryotic strains including PMS-45 belong to the *B1* mating-type, and the other three monokaryotic strains including PMS-67 are the *B2* mating-type.

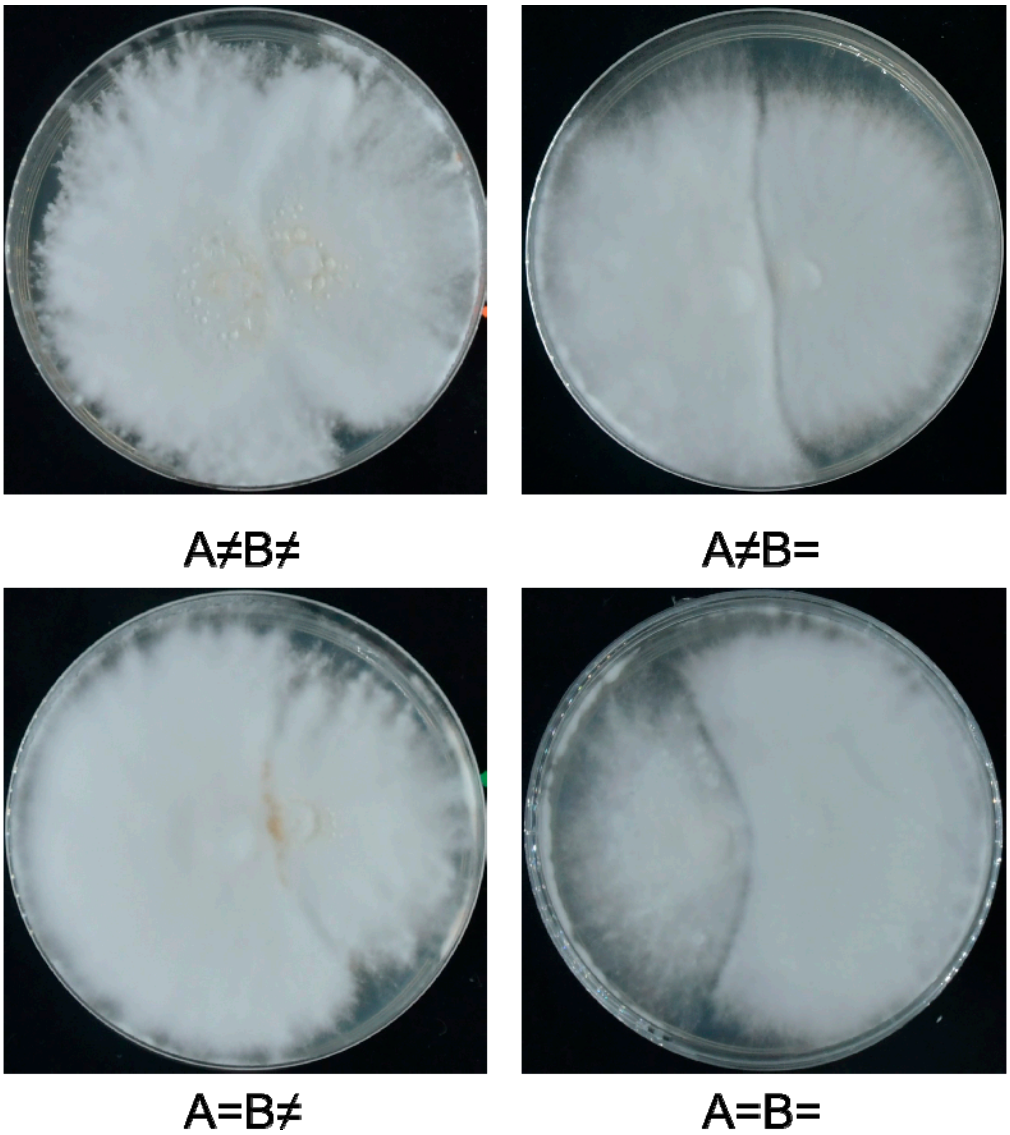


**Figure S4.** Antagonism test of the monokarytic strains of different mating-type. The symbol A≠B≠ indicates that both the *A* and *B* mating-type differ between strains; A=B= means the *A* and *B* mating-type between strains are identical.

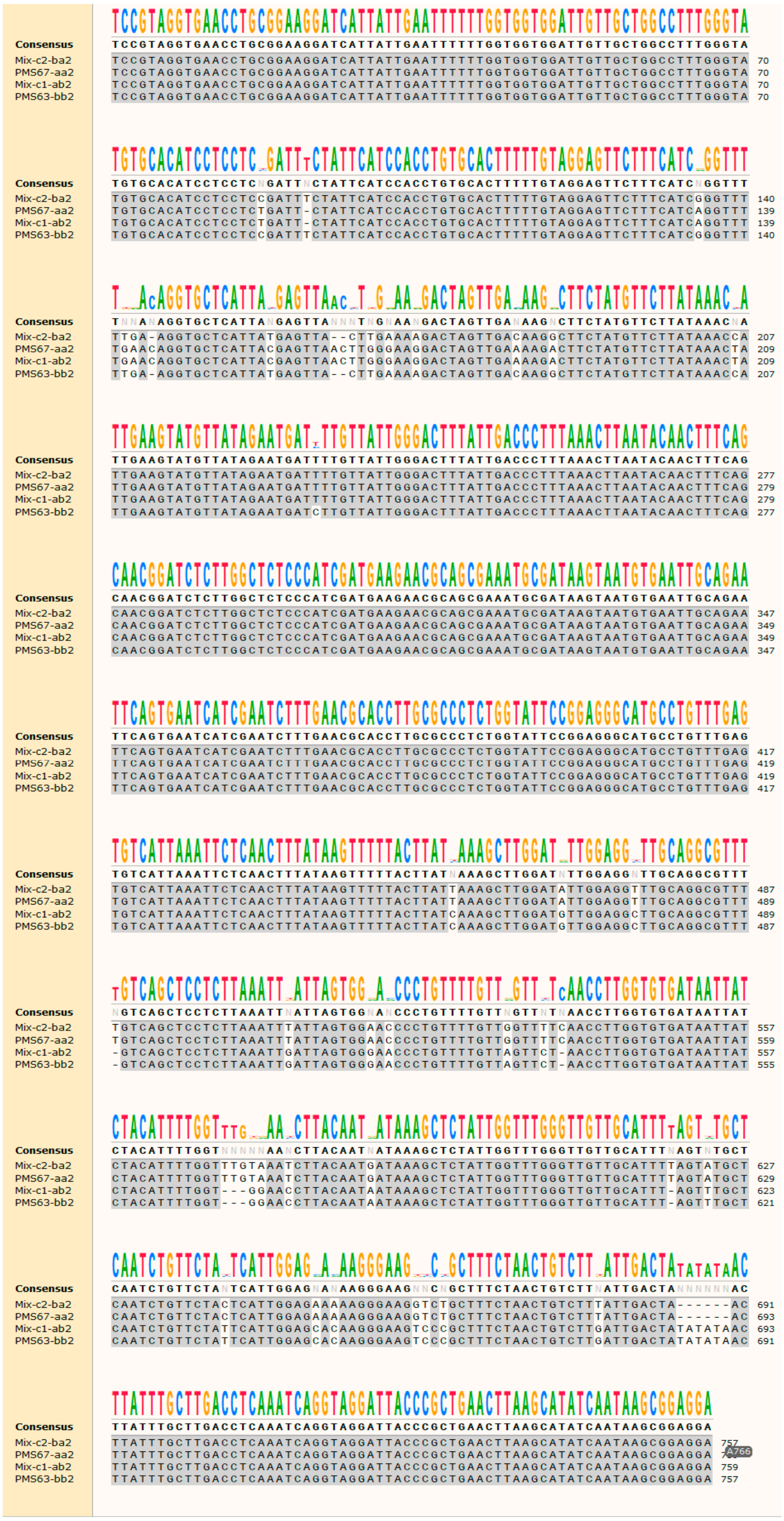

**Figure S5.** Sequence alignments of the chimeric amplicons from mix template. Mix-c1-ab2: chimeric ITS-ab2 sequence generated by PCR, cloning and sequencing from an equal DNA mixture of strains PMS67 and PMS63; Mix-c2-ba2: another ITS-ba2 type. chimeric sequence.

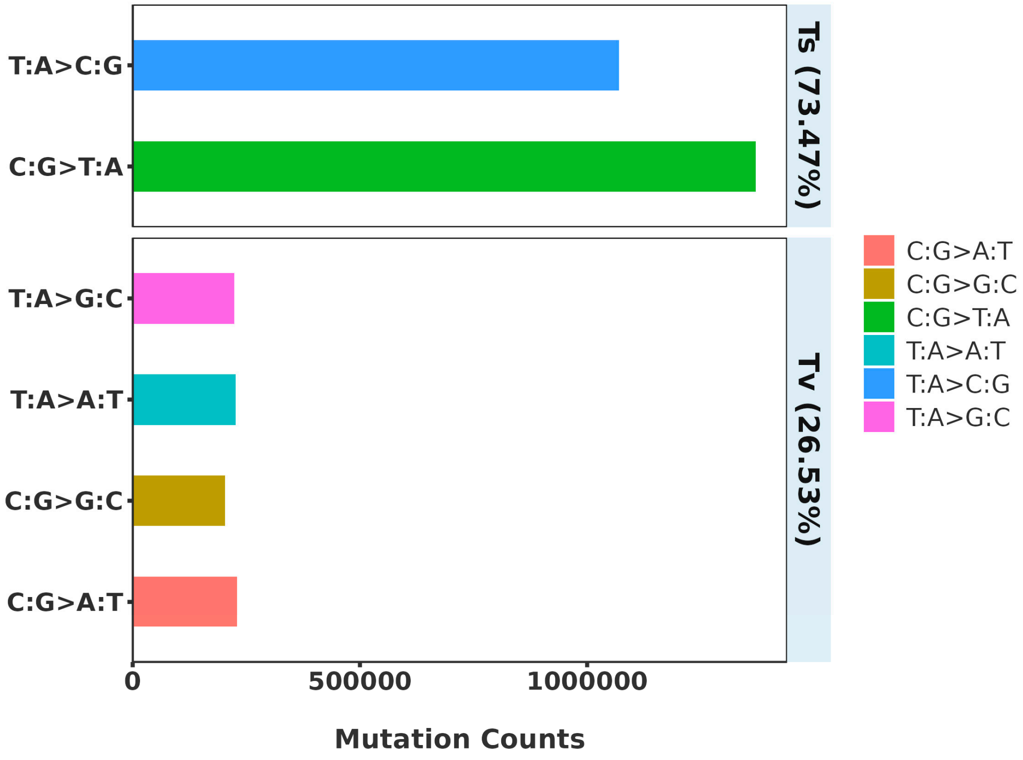


**Figure S6.** Analyses of SNP mutation spectrum. T: A > C: G and C :G > T: A represent the major SNP variants.

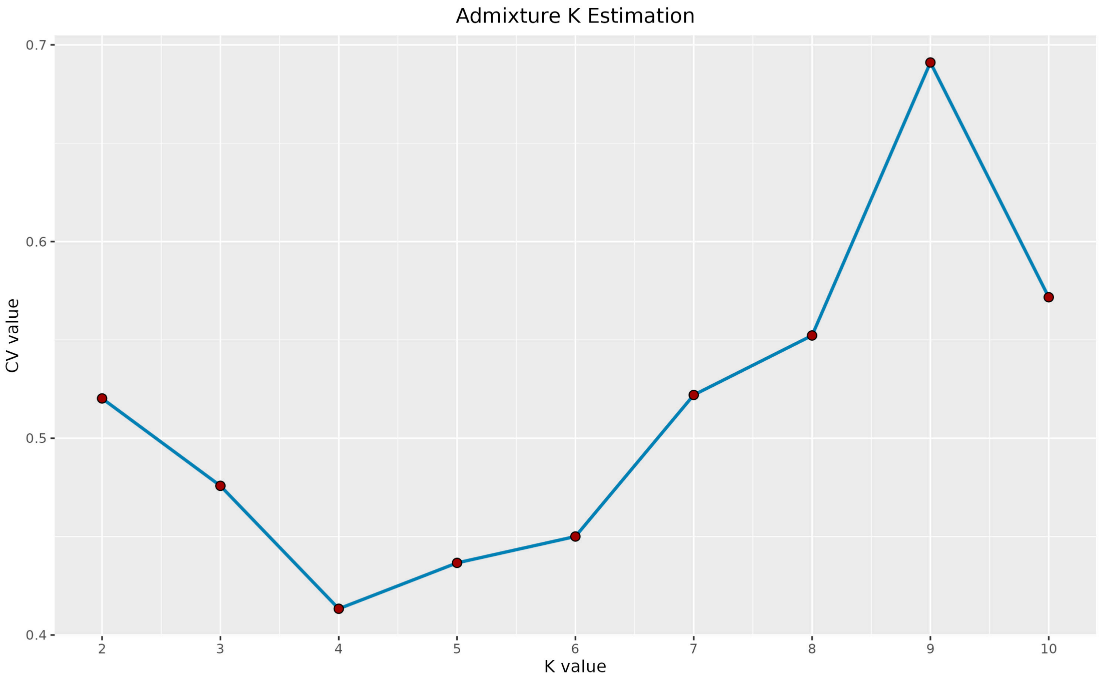


**Figure S7.** Distribution map of CV error values to different K values. The CV values reach the minimum when K=4, corresponding to the optimal value for the number of subpopulations.

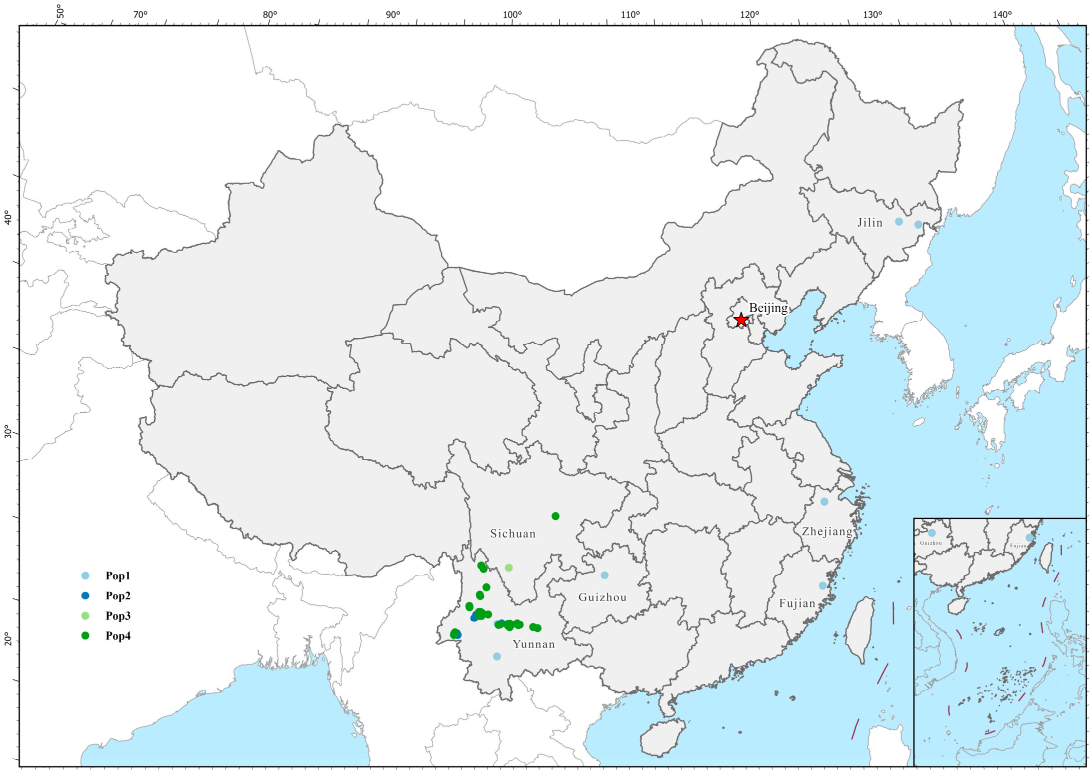


**Figure S8.** Distribution map of 50 *L.edodes* strains from four populations. Dots in different colors represent the populations to which the strains belong.
